# Incremental Value of a Panel of Serum Metabolites for Predicting Risk of Atherosclerotic Cardiovascular Disease

**DOI:** 10.1161/JAHA.121.024590

**Published:** 2022-02-12

**Authors:** Ana Nogal, Panayiotis Louca, Tran Quoc Bao Tran, Ruth C. Bowyer, Paraskevi Christofidou, Claire J. Steves, Sarah E. Berry, Kari Wong, Jonathan Wolf, Paul W. Franks, Massimo Mangino, Tim D. Spector, Ana M. Valdes, Sandosh Padmanabhan, Cristina Menni

**Affiliations:** ^1^ Department of Twin Research and Genetic Epidemiology King’s College London London United Kingdom; ^2^ Institute of Cardiovascular & Medical Sciences University of Glasgow Glasgow United Kingdom; ^3^ Department of Nutritional Sciences King’s College London London United Kingdom; ^4^ Metabolon Morrisville NC; ^5^ Zoe Limited London United Kingdom; ^6^ Department of Clinical Sciences Lund University Diabetes Center Lund University Malmö Sweden; ^7^ Harvard T. H. Chan School of Public Health Boston MA; ^8^ NIHR Biomedical Research Centre at Guy’s and St. Thomas’ Foundation Trust London United Kingdom; ^9^ Injury, Recovery and Inflammation Sciences Clinical Sciences Building Nottingham City Hospital, University of Nottingham Nottingham United Kingdom

**Keywords:** atherosclerosis, biomarkers, cardiovascular disease risk, machine learning, serum metabolites, Epidemiology, Cardiovascular Disease, Atherosclerosis

Cardiovascular diseases (CVDs) are the leading causes of mortality and morbidity worldwide, accounting for 17.3 million deaths per year.^1^ The American College of Cardiology/American Heart Association 10‐year atherosclerotic CVD risk score is a sex‐ and race‐specific single multivariable risk assessment tool used to estimate the 10‐year CVD risk of an individual based on age, sex, and traditional risk factors (TRFs), including high‐density lipoprotein and total cholesterol, blood pressure, blood pressure medications, smoking, and type 2 diabetes.^1^ These factors contribute considerably to disease risk, although they may not identify at‐risk individuals before disease onset.^2^, ^3^ Previous studies found circulating metabolites predictive of cardiovascular traits, mostly using linear approaches and a limited number of metabolites.^3^, ^4^, ^5^

By combining the effects of a larger number of individual biomarkers, TRFs, and environmental variables, we applied a machine learning technique to identify a metabolite panel cross‐sectionally associated with estimated atherosclerotic CVD (eASCVD) risk and longitudinally predictive of CVD mortality and morbidity in a population‐based cohort with independent replication, to gain further insights into the metabolic pathways underlying CVD risk.

The data used in this study are held by the Department of Twins Research at King’s College London. The data can be released to bona fide researchers using our normal procedures overseen by the Wellcome Trust and its guidelines as part of our core funding (https://twinsuk.ac.uk/resources‐for‐researchers/access‐our‐data/). The scripts in R and all the necessary information to replicate the findings reported in this article are publicly available at https://github.com/ananogal1/ASCVD‐metabolite‐panel.

The flowchart of the study design is depicted in the Figure (A). We included women from TwinsUK^1^ with fasting serum metabolomic profiling (533 metabolites; Metabolon) along with eASCVD,^1^ TRFs, diet (healthy eating index),^1^ menopause status, and physical activity at 2 time points 6 years apart (SD=2) (Figure [B]). Individuals with prevalent CVD were excluded. TwinsUK provided informed written consent, and the study was approved by the St. Thomas’ Hospital Research Ethics Committee (REC Ref: EC04/015).

Metabolites were inverse normalized, and missing values imputed using minimum run‐day measures. For each metabolite, we calculated residuals by running linear regressions adjusting for age, body mass index, menopause status, diet, and physical activity. To identify a metabolite panel associated with eASCVD, we built random forest models on the residuals at each time point, splitting the data set into training and test sets (80:20). We tuned hyperparameters using the adaptive resampling search and used 5‐fold cross‐validation and node purity to select the optimal predictors’ number. We identified common predictors between the 2 time points and examined the effect on model prediction using the Shapley additive explanations plot. Common metabolites with concordant effects at both time points were included in the eASCVD metabolites panel. Results were replicated in 295 women from PREDICT‐1 (Personalised Responses to Dietary Composition Trial).^1^ We further tested the incremental area under the curve (AUC) value of the eASCVD metabolites panel in predicting incident cardiac disease (including congestive heart disease, angina, atrial fibrillation, and coronary heart disease) and CVD mortality (through record linkage with the Office for National Statistics [ONS]) in independent sets of 50 to 134 individuals (follow‐up, 5.6 years [SD, 2.2 years]). Finally, we explored the pathways in which the identified metabolites were involved using Ingenuity Pathway Analysis (QIAGEN; Fisher exact test, false discovery rate [Benjamini‐Hochberg] <0.05).

The random forest models on residuals in 1066 TwinsUK women adjusted for age, body mass index, menopause, physical activity, and diet identified 100 and 67 predictors of eASCVD at time point 1 and 2, respectively, of which 25 were overlapping. Of these, 21 had concordant effects at both time points and were included in the eASCVD metabolites panel. After adjusting for family, the panel explained 12.7% of the variance in eASCVD in the test set and 13.6% in PREDICT‐1. When further adjusting for TRFs, the panel explained 9.3% in the test set and 8.5% in PREDICT‐1. Among the metabolites identified, 9 were positively associated with eASCVD, whereas 12 were negatively associated (Figure [C]). The peptide phenylalanyltryptophan, the lipid choline phosphate, and the amino acid 4‐hydroxyphenylpyruvate were the most important contributors (Figure [C]). The incremental predictive value of the eASCVD‐metabolites panel over environmental and TRFs improved prediction of incident cardiac disease by 7% (AUC from 0.68 [95% CI, 0.57–0.78] to 0.75 [95% CI, 0.66–0.88]) and CVD mortality by 4% (AUC from 0.68 [95% CI, 0.62–0.91] to 0.72 [95% CI, 0.67–0.96]) (Figure [D]). Finally, pathway enrichment analysis highlighted the involvement (false discovery rate range = 0.01–0.02) of the metabolites positively associated with eASCVD in the biosynthesis of 4‐hydroxyphenylpyruvate, choline, phosphatidylcholine and glucocorticoids, sphingomyelin metabolism, tyrosine degradation, and phospholipases. Moreover, the panel was enriched (false discovery rate range = 0.001–0.04) in metabolites related to cardiac inflammation, dysfunction damage, and infarction.

Here, we report for the first time a panel of serum metabolites correlated with eASCVD explaining 9.3% of the variance not already explained by environmental and TRFs. The panel further improved prediction of incident cardiac disease and CVD mortality over and above conventional risk factors, thereby generating new research avenues. Metabolites positively associated with eASCVD are enriched in pathways previously linked with atherosclerotic CVD.^2^ The sphingomyelin:phosphatidylcholine ratio, choline and glucocorticoids biosynthesis, tyrosine degradation, and phospholipases have been shown to increase the CVD risk and/or mortality risk.^2^, ^4^, ^5^ Therefore, this study sheds light into the metabolites behind these pathways.

**Figure 1:**
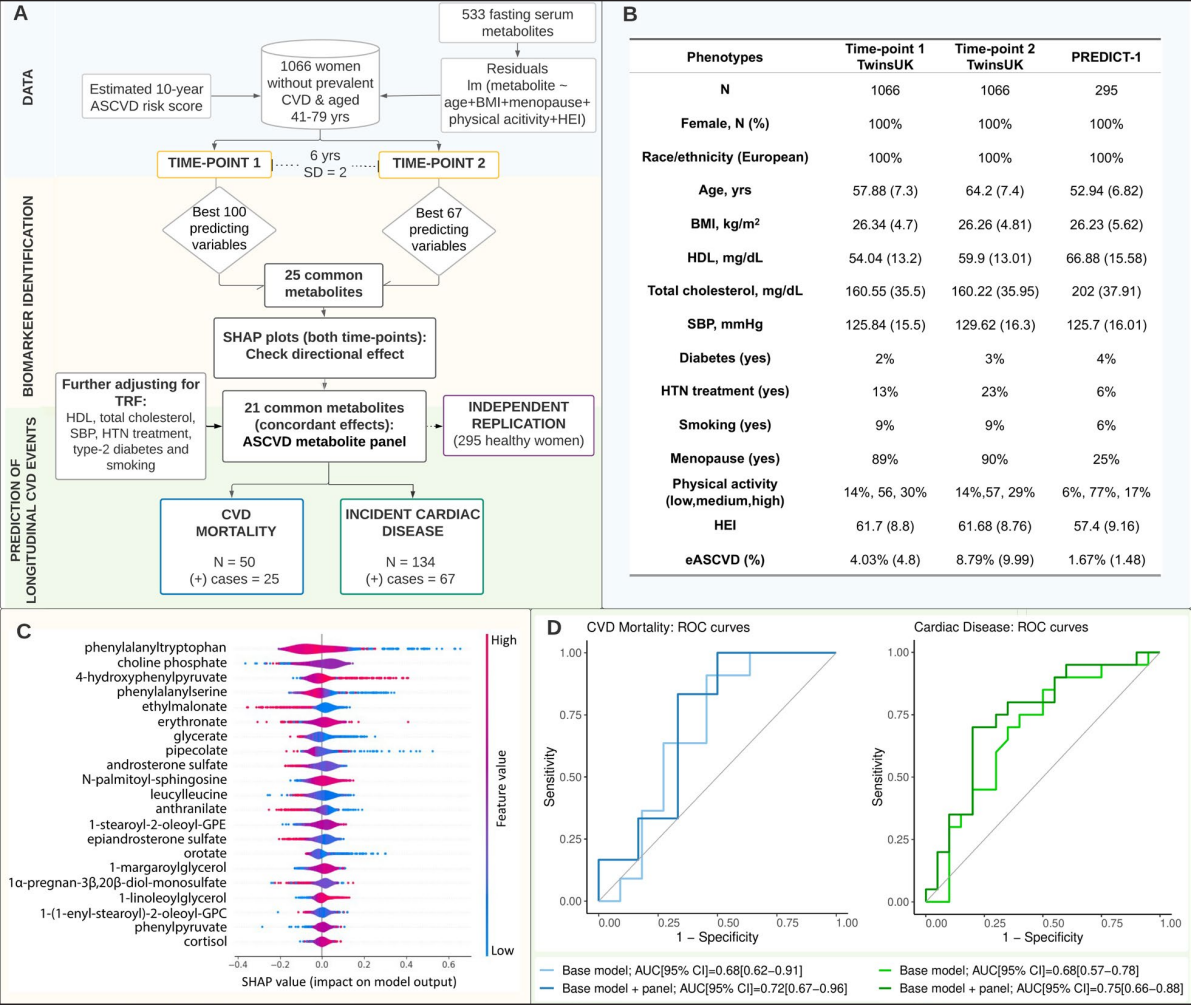
Serum metabolites associated to atherosclerotic cardiovascular disease: flowchart, data, and main results. **A**, Flowchart of the study design. "N" indicates the number of individuals included to build the random forest (RF) classifiers, whereas "(+) cases" refers to the number of individuals suffering from a specific cardiovascular disease (CVD) phenotype. **B**, Demographic characteristics of the study samples PREDICT‐1 (TwinsUK and Personalised Responses to Dietary Composition Trial). Demographic characteristics by outcome (ie, incident cardiac disease and CVD mortality) are provided on GitHub. **C**, Directional effect of each single metabolite from the estimated atherosclerotic cardiovascular disease (eASCVD) risk panel on the model predictions using a Shapley additive explanations (SHAP) plot. The SHAP values (*x* axis) quantify the magnitude and direction (positive or negative using the feature values) of each metabolite on the target variable (ASCVD). Each point represents a feature instance, whereas the color indicates the feature value (high=red, low=blue). **D**, Area under the curve (AUC) values and receiver operating characteristic (ROC) curves obtained for RF classifiers built on (1) the base model including environmental and traditional risk factors and (2) the base model plus the eASCVD metabolites panel. Each ROC curve represents the performance of the RF classifiers in predicting each CVD event (CVD mortality and incident cardiac disease) at different classification thresholds (range = 0–1). The AUC is computed for each curve and used as a model performance metric. ASCVD indicates atherosclerotic cardiovascular disease; BMI, body mass index; GPC, glycerophosphocholine; GPE, glycerophosphoethanolamine; HEI, health eating index; HDL, high‐density lipoprotein; HTN, hypertension; lm, linear models; SBP, systolic blood pressure; and TRF, traditional risk factors.

Limitations include the homogeneous ethnicity and women‐only composition of the samples, the lack of longitudinal data in PREDICT‐1, and the limited number of CVD events. However, we benefit from cross‐sectional ASCVD data, independent data sets to test the panel predictive power, and independent replication. Our results illustrate how metabolic profiling along with machine learning might identify novel biomarkers implicated in CVD, which are crucial for early diagnosis and treatment.

## Sources of Funding

This research was funded by the Chronic Disease Research Foundation and in part by the Wellcome Trust (grant number: 212904/Z/18/Z). For the purpose of open access, the authors have applied a Creative Commons Attribution (CC BY) public copyright license to any author‐accepted article version arising from this submission. TwinsUK receives funding from the Wellcome Trust, the European Commission H2020 grants SYSCID (contract number 733100); the National Institute for Health Research (NIHR) Clinical Research Facility and the Biomedical Research Centre based at Guy's and St. Thomas' National Health Service (NHS) Foundation Trust in partnership with King's College London, the Chronic Disease Research Foundation, the UK Research and Innovation (UKRI) Medical Research Council (MRC)/British Heart Foundation, Ancestry and Biological Informative Markers for Stratification of Hypertension (AIM‐HY) (MR/M016560/1), and Zoe Limited. Dr Mangino is supported by the NIHR Clinical Research Facility and the Biomedical Research Centre based at Guy's and St. Thomas' NHS Foundation Trust in partnership with King's College London. Dr Menni is funded by the Chronic Disease Research Foundation and by the UKRI MRC AIM‐HY project grant (MR/M016560/1). A. Nogal and P. Louca are funded by the Chronic Disease Research Foundation. Dr Valdes is supported by the National Institute for Health Research Nottingham Biomedical Research Centre. Dr Christofidou is funded by the European Union (H2020 contract number 733100). Dr Franks receives support from the Swedish Research Council, Swedish Heart‐Lung Foundation and the Swedish Foundation for Strategic Research (LUDC‐IRC 15‐0067). Dr Padmanabhan is funded by the UKRI MRC AIM‐HY project grant (MR/M016560/1); the British Heart Foundation (BHF) (CS/16/1/31878) and BHF Centre of Excellence (RE/18/6/34217). The PREDICT cohort is supported by Zoe Limited.

## Disclosures

Drs Valdes, Franks, and Berry are consultants to Zoe Limited, and J. Wolf and Dr Spector are cofounders of Zoe Limited. Dr Wong is an employee of Metabolon Inc. All other authors declare no competing financial interests.

## References

[1] Berry SE , Valdes AM , Drew DA , Asnicar F , Mazidi M , Wolf J , Capdevila J , Hadjigeorgiou G , Davies R , Al Khatib H , et al. Human postprandial responses to food and potential for precision nutrition. Nat Med. 2020;26:964–973. doi: 10.1038/s41591-020-0934-0 32528151 PMC8265154

[2] Ussher JR , Elmariah S , Gerszten RE , Dyck JR . The emerging role of metabolomics in the diagnosis and prognosis of cardiovascular disease. J Am Coll Cardiol. 2016;68:2850–2870. doi:10.1016/j.jacc.2016.09.972 28007146

[3] Wang Z , Zhu C , Nambi V , Morrison AC , Folsom AR , Ballantyne CM , Boerwinkle E , Yu B . Metabolomic pattern predicts incident coronary heart disease: findings from the atherosclerosis risk in communities study. Arterioscler Thromb Vasc Biol. 2019;39:1475–1482. doi: 10.1161/ATVBAHA.118.312236 31092011 PMC6839698

[4] Murthy VL , Reis JP , Pico AR , Kitchen R , Lima JAC , Lloyd‐Jones D , Allen NB , Carnethon M , Lewis GD , Nayor M , et al. Comprehensive metabolic phenotyping refines cardiovascular risk in young adults. Circulation. 2020;142:2110–2127. doi: 10.1161/CIRCULATIONAHA.120.047689 33073606 PMC7880553

[5] Cavus E , Karakas M , Ojeda FM , Kontto J , Veronesi G , Ferrario MM , Linneberg A , Jørgensen T , Meisinger C , Thorand B , et al. Association of circulating metabolites with risk of coronary heart disease in a European population: results from the Biomarkers for Cardiovascular Risk Assessment in Europe (BiomarCaRE) Consortium. JAMA Cardiol. 2019;4:1270–1279. doi: 10.1001/jamacardio.2019.4130 31664431 PMC6822093

